# The prevalence and correlation of cancer-related fatigue and locomotive syndrome in geriatric cancer patients

**DOI:** 10.1371/journal.pone.0319511

**Published:** 2025-03-10

**Authors:** Mo-Yu Yang, Ying Chen, Hui Su, Yan Lv, Yu-Ling Yang

**Affiliations:** 1 Department of Oncology, Affiliated Hospital of Jiangnan University, Wuxi, Jiangsu, China; 2 Department of Medical check-up center, Taihu Sanatorium of Jiangsu Province, Wuxi, Jiangsu, China; University of Toronto Temerty Faculty of Medicine, CANADA

## Abstract

**Objective:**

To explore the relationship between cancer-related fatigue (CRF) and locomotive syndrome (LS) among Chinese older adults with cancer.

**Methods:**

This study was a cross-sectional survey. Staged random sampling method was employed to enroll 500 geriatric cancer patients from the Oncology Center of Jiangnan University Hospital in Wuxi, Jiangsu Province, China. Data were collected using General Information Questionnaire, Cancer Fatigue Scale and the Geriatric Locomotive Function Scale. *χ*^2^ test, Logistic regression analysis and Spearman analysis were utilized to analyze the data.

**Results:**

A total of 466 geriatric cancer patients were enrolled in this survey. The occurrence of CRF was 43.8%, and the occurrence of LS was 30.9%. LS were risk factors for the occurrence of CRF in geriatric cancer patients. Advanced old age, fear of falling, comorbid with chronic diseases and CRF were risk factors for LS. CRF was positively associated with LS (*r* = 0.446, *P* < 0.001).

**Conclusion:**

Our findings indicate a potential link between CRF and LS. By addressing LS from various aspects, including controlling the number of complications due to advanced old age and other factors, as well as spreading proper fall-related knowledge and controlling the awareness of fear of falling, healthcare professionals can indirectly alleviate CRF symptoms and improve the overall quality of life for these patients.

## Introduction

Global epidemiological data on cancer deaths indicate that by the end of 2020, there were 9.96 million cancer-related deaths worldwide, 3 million of which were from China, accounting for 30% of the total deaths [1]. Available data show that majority of cancer patients in China are elderly [2]. This group is expected to expand significantly in the coming decades driven by the evolving pattern of social life and medical technology [2]. For geriatric cancer patients, their advanced age often means they have multiple existing health conditions. Additionally, the often-intense treatments for their cancer can cause a variety of side effects. These factors, combined with the direct effects of the tumor itself, have a significant negative impact on their overall quality of life (QoL) [3].

Cancer-related fatigue (CRF) is one of the more common adverse reactions and refers to a long-lasting, subjective feeling of exhaustion driven by somatic, emotional, and cognitive factors. The severity of CRF does not correspond to the patient’s level of activity [4,5]. CRF not only reduces patients’ quality of life, but also increases the occurrence of depression, anxiety, and other adverse moods [6]. Modern medicine recognizes that improving a patient’s symptoms and QoL during cancer treatment is just as crucial as controlling the disease itself. This growing focus has led medical practitioners to place greater emphasis on managing CRF alongside treatment [7]. Previous studies on geriatric cancer patients have not focused on the CRF status. Given that these patients have relatively complex conditions and more complications, early screening and identification of CRF in geriatric cancer patients should be enhanced [8].

Successful rehabilitation for musculoskeletal function (MF) depends on timely recognition and intervention. Locomotive syndrome (LS) describes a condition where weakness or damage to the muscles, bones, and joints increases the difficulty of walking, standing, and other movements. This can lead to a current or future need for assistance with daily activities [9]. The progression of LS is accompanied by several symptoms including pain, limited range of motion in joints, poor posture, and imbalance, which in causes difficulties in standing and walking, deterioration of MF, decreased ability of daily living (ADL) and QoL, which may be fatal and necessitate times intervention [10]. Unlike conditions like sarcopenia and debilitation that target specific symptoms, LS takes a broader approach, assessing an individual’s overall MF [9]. This is crucial because the gradual decline in muscles, bones, and other locomotor organs associated with LS can be slow and subtle, making early detection difficult [11]. Based on this, Kawano concluded that LS is a more sensitive indicator for detecting MF disorders, and timely prevention and improvement of LS status are key to maintaining ADL and QoL in oncology patients [12].

LS may have a direct or indirect effect on CRF by limiting the patient’s ability to exercise, exacerbating pain, and causing psychological distress, but the specific association between CRF and LS has not been fully explored [12,13]. In this study, we investigated and analyzed the prevalence of CRF and LS in geriatric cancer patients and explored their correlations. This study will provide reference data for preventing and slowing down the occurrence of CRF and LS in geriatric cancer patients.

By identifying the association between CRF and LS in geriatric cancer patients, clinicians can detect high-risk patients earlier and implement targeted interventions to prevent further functional decline. Additionally, this research addresses a gap in the literature concerning the relationship between CRF and LS in geriatric cancer patients, providing new theoretical insights into functional decline in this demographic. This improves the quality of life for geriatric cancer patients and provides a new direction for developing multidisciplinary comprehensive treatment plans.

## Materials and methods

This study employed a single-center cross-sectional study design. The study was conducted according to the guidelines of the Declaration of Helsinki, and approved by the Ethics Committee of the Affiliated Hospital of Jiangnan University (No. LS2023101) and completed the Chinese Clinical Trial Registration (No. ChiCTR2400079958), the date of first registration is 17/01/2024. Informed consent was obtained from all subjects involved in the study. Written informed consent has been obtained from the patients to publish this paper.

### Objects

A total of 500 geriatric cancer patients who were treated at the Oncology Center of the Affiliated Hospital of Jiangnan University in Wuxi, Jiangsu Province, China, were enrolled using a convenience sampling method from January 2024 to March 2024.

**Inclusion criteria:** ① ≥ 60 years old; ② Confirmed diagnosis of malignant tumors according to the 8th edition of the International Tumor TNM Staging Criteria, and they had no bone metastases; ③ Receiving active treatments for tumor chemotherapy and other therapies, not palliative treatments; ④ Barthel Index > 60; ⑤ Had no history of MF disorder caused by the primary disease of the orthopedic system; ⑥ Voluntarily provided informed consent to participate.

**Exclusion criteria:** ① Any contraindication to exercise; ② Serious physical or mental illness, such as depression; ③ Cognitive dysfunction.

### Research tools

#### General information questionnaire.

The questionnaire collected participant information on demographics (gender, age, marital status, education level), socioeconomic factors (average annual household income, residence), and health history (history of falling, fear of falling, number of comorbid chronic diseases). Comorbid chronic diseases are defined as patients who have a cancer along with one or more chronic diseases. In this study, chronic diseases included hypertension, diabetes mellitus, dyslipidemia, stroke or chronic respiratory diseases with clear medical diagnosis.

To ensure the accuracy and consistency of the data, we implemented the following standardized approach to inquire about fall history and fear of falling:

Question design: “Have you experienced a fall in the past six months? (Yes/No)”/ “How concerned are you about the risk of falling?”/ “Do you avoid certain activities or behaviors due to the fear of falling?”

We provided specific response options for patients to select based on their personal experiences, ensuring that the questions were simple and clear to avoid any potential biases arising from language or comprehension issues.

#### Barthel Index.

The Barthel Index was developed by Dorother Barthel and Floorence Mahney in 1965 to assess the ability of subjects to perform specific tasks in their daily lives, including eating, bathing, dressing, bowel control, urination control, toileting, walking on flat surfaces, walking up and down stairs, and transferring from one bed to another. The scale consists of 10 items, including eating, bathing, dressing, bowel control, urinary control, toilet use, walking on level ground, walking up and down stairs, and bed and chair transfers, and is scored out of a total of 100 points, with higher scores indicating greater self-care. Barthel Index > 60 means the participant has mild functional impairment but is basically living on their own. The Cronbach’s α coefficient for the scale was 0.762 [14].

#### Cancer fatigue scale, CFS.

The CFS was compiled by Okuyama et al., to measure CRF in patients with malignant tumors [15]. It contains 15 entries in 3 dimensions, namely, somatic fatigue (7 entries), cognitive fatigue (4 entries), and affective fatigue (4 entries), with each entry counting from 0 to 4 from not at all, rarely, a little, quite a bit, and very much, respectively, and a total score of 0-60, with the higher scores indicating a higher degree of fatigue. A score > 18 indicated a CRF. The Cronbach’s α coefficient for the scale was 0.925.

#### The geriatric locomotive function scale, GLFS-25.

The GLFS-25 developed by Seichi et al. was used to measure locomotive syndrome in the elderly [16]. The scale has 4 dimensions of pain (4 entries), activities of daily living (16 entries), social functioning (3 entries), and mental health (2 entries), with a total of 25 entries. Each entry was assigned a score from 0 to 4 in the order of no difficulty - very difficult, with higher scores representing poorer motor function. The Cronbach’s *α* of the scale was 0.927, and a score of ≥ 16 indicated that the subject had LS.

#### Data collection process.

Before the survey, we contacted the head of the oncology center and obtained permission for recruitment of eligible patients after obtaining informed consent from elderly oncology patients. The investigators broadcasted the message and distributed the questionnaires centrally in the conference room of the oncology center to the eligible patients. Before administering the survey, the researchers underwent standardized training to ensure consistent instructions. They then approached elderly oncology patients, explained the survey’s purpose and methodology, and obtained informed consent. The survey was conducted face-to-face, with each patient completing the questionnaire independently whenever possible. For patients unable to complete the questionnaire on their own, a researcher assisted by recording their responses accurately without using suggestive prompts. A total of 500 questionnaires were distributed and collected on the spot, and 466 valid questionnaires were retrieved, corresponding to an effective recovery rate of 93.2%.

#### Statistical analysis.

EpiData 3.1 was utilized to create a database, and all information was entered and checked by two people on two machines. Statistical analysis was conducted using the SPSS 25.0 software. Qualitative information was described by frequency and percentage, and analyzed by *χ*^2^ test. Logistic regression analysis was performed to analyze the risk factors of CRF and LS. Spearman analysis was employed to determine the correlation between CFS scores and GLFS-25 scores. The test level was set at *α* =  0.05.

## Results

### Basic information about the geriatric cancer patients

**Fig 1:**
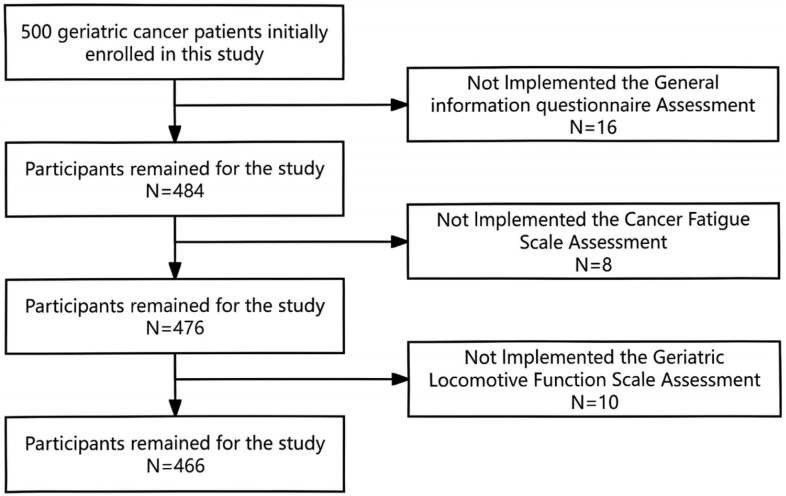
Flowchart of the subject recruitment process.

**Fig 2:**
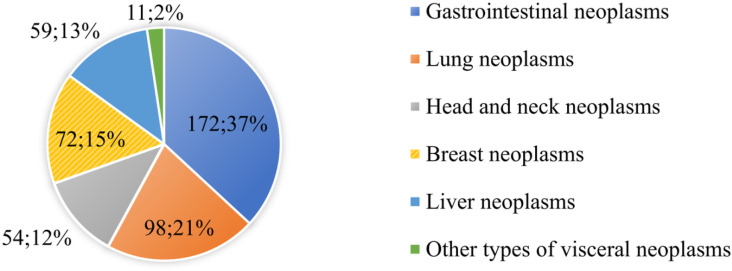
The origin of the malignant cancer of 466 geriatric cancer patients.

A total of 500 Chinese geriatric cancer survivors were initially enrolled in the study. After the removal of incomplete questionnaires, 466 valid ones were retained, reflecting a response rate of 93.2% (Fig.1). The average old of the enrolled patients was 74.1 ± 10.0 years old; 207 males and 259 females. The average time since first cancer diagnosis was 4.3 ± 2.7 years. The mean GLFS-25 score was 13.6 ± 6.5. The distribution of origin of malignant cancer is presented in Fig.2.

#### Occurrence of CRF and LS in geriatric cancer patients.

LS occurred in 144 (30.9%) and CRF was recorded in 204 (43.8%) of geriatric cancer patients. There were differences in characteristics of occurrence of CRF and LS in geriatric cancer patients as shown in Table 1. As shown in Table 1, there was a difference in CRF status among geriatric cancer patients with different gender, literacy level, history of falling, fear of falling, history of cancer metastasis, history of cancer-related surgeries, history of radiotherapy, history of chemotherapy, history of cancer recurrence, and LS status (*P* < 0.05). LS status varies among geriatric cancer patients with different gender, age, marital statuses, literacy levels, history of falling, fear of falling, number of comorbid chronic diseases, history of cancer metastasis, history of cancer-related surgeries, history of chemotherapy, history of cancer recurrence, and CRF status (*P* < 0.05).

**Table 1.** Comparison of LS and CRF occurrence in geriatric cancer patients with different characteristics.

Variables	Classification	N	CRF			LS		
			N	*χ* ^*2*^	*P*	N	*χ* ^*2*^	*P*
Gender	Male	207	111	6.823	0.009*	53	5.903	0.015*
	Female	259	93			91		
Age	60 ~ 69	308	73	3.083	0.358	81	8.637	0.013*
	70 ~ 79	128	33			48		
	80 ~ 90	30	98			15		
Marital status	Married	356	182	0.463	0.476	99	4.457	0.021*
	Other status	110	22			45		
Literacy level	Junior high school and below	337	116	8.618	0.033*	86	7.663	0.029*
	Senior high school and above	159	88			58		
Residence	Live alone	41	22	0.486	0.587	18	2.132	0.278
	With co-residents	425	182			126		
History of falling	None	374	133	5.044	0.026*	96	8.631	0.013*
	Yes	92	71			48		
Fear of falling	None	192	60	5.386	0.024*	34	31.467	<0.001*
	Yes	274	144			110		
Comorbid chronic diseases†	0	128	56	9.486	0.064	16	33.407	<0.001*
	1	166	53			28		
	2	117	65			26		
	≥3	55	30			74		
History of cancer metastasis	None	238	40	10.345	<0.001*	53	8.342	<0.001*
	Yes	228	164			91		
History of cancer-related surgeries	None	150	72	5.423	0.036*	28	6.439	0.034*
	Yes	316	132			116		
History of radiotherapy	None	378	162	4.044	0.046*	104	3.452	0.514
	Yes	88	42			40		
History of chemotherapy	None	50	12	5.223	0.036*	13	6.453	0.032*
	Yes	416	192			131		
History of cancer recurrence	None	406	176	5.345	0.043*	116	4.563	0.036*
	Yes	60	28			28		
CRF	None	262				48	36.884	<0.001*
	Yes	204				96		
LS	None	322	108	36.881	<0.001*			
	Yes	144	96					

†:Including hypertension, diabetes mellitus, dyslipidemia, stroke or chronic respiratory diseases with clear medical diagnosis.

*: P < 0.05

### Multifactorial analysis of factors influencing the occurrence of CRF and LS in geriatric cancer patients

#### Multifactorial analysis of the occurrence of CRF.

Logistic regression analysis was conducted to identify factors influencing the occurrence of CRF in geriatric cancer patients. The analysis included variables identified as statistically significant (*P* < 0.05) in the *χ*^2^ analysis as independent variables, with CRF as the dependent variable. The results revealed that LS were the main factors associated with CRF in this population (*P* < 0.05). See Table 2 for detailed information.

**Table 2.** Logistic regression analysis of factors influencing CRF in geriatric cancer patients.

Variables	*β*	*SE*	Wald *χ*^2^	*P*	*OR*	95%*CI*
Constant	-2.802	0.412	46.215	<0.001	0.012	–
Literacy level (Junior high school and below as reference)						
Senior high school and above	-0.403	0.384	1.102	0.296	0.668	0.314 ~ 1.421
LS (None as reference)						
Yes	1.209	0.272	19.728	<0.001	3.351	1.966 ~ 5.714

Assignment of variables: Literacy level: Junior high school and below =  1, Senior high school and above =  2; LS: None =  0, Yes =  1;

#### Multifactorial analysis of the occurrence of LS.

Logistic regression analysis was employed to investigate the factors associated with LS in geriatric cancer patients. The analysis included LS as the dependent variable and variables that manifested statistically (P < 0.05) significant disparities in the *χ*^2^ test as independent variables. The results showed that age, fear of falling, comorbid chronic diseases and CRF were risk factors for occurrence of LS in geriatric cancer patients (*P* < 0.05). See Table 3 for detailed information.

**Table 3.** Logistic regression analysis of factors influencing LS in geriatric cancer patients.

Variables	*β*	*SE*	Wald χ^2^	*P*	*OR*	95%*CI*
Constant	-3.100	0.503	37.948	<0.001		
Age (60-69 as reference)						
70 ~ 79	0.559	0.281	3.969	0.046	1.749	1.009 ~ 3.032
80 ~ 90	1.311	0.610	4.614	0.032	3.709	1.122 ~ 12.266
Fear of falling (None as reference)						
Yes	1.070	0.278	14.836	<0.001	2.916	1.692 ~ 5.027
Comorbid chronic diseases (0 as reference)						
1	0.628	0.424	2.193	0.139	1.874	0.816 ~ 4.301
2	0.486	0.448	1.179	0.278	1.626	0.676 ~ 3.913
≥3	1.291	0.395	10.688	0.001	3.637	1.677 ~ 7.886
CRF (None as reference)						
Yes	1.187	0.279	18.123	<0.001	3.278	1.898 ~ 5.662

Assignment of variables: Age: 60-69 =  1, 70-79 =  2, > 79 =  3; FoF: None =  0, Yes =  1; Education level: Junior high school and below =  1, Senior high school and above =  2; Comorbid chronic diseases: 0 =  1, 1 =  2, 2 =  3, ≥ 3 =  4; CRF: None =  0, Yes =  1.

### Correlation between CRF and LS in geriatric cancer patients

Analysis of the Spearman’s correlation results showed that the total GLFS-25 score and four dimensions were positively correlated with CRF in geriatric cancer patients as presented in Table 4.

**Table 4.** Correlation of GLFS-25 score with CRF score.

Variables	*R*	*P*
GLFS-25	0.446	<0.001
Pain	0.297	<0.001
Daily living activities	0.331	<0.001
Social function	0.320	<0.001
Mental health	0.368	<0.001

## Discussion

### Occurrence of CRF and risk factors in geriatric cancer patients

CRF is a common and persistent symptom in cancer patients and is one of the most distressing symptoms [17]. CRF negatively affects the treatment, recovery and body function of patients, and there is no clear and effective medication to control it [18]. In this study, we found that the occurrence of CRF in elderly tumor patients was 43.8%, and multivariate logistic regression analysis showed that literacy and LS were the main risk factors for CRF in elderly tumor patients while controlling for other factors (P < 0.05).

Notably, findings from prior studies on the effect of educational level on CRF are inconclusive. Some studies have found no direct correlation between education and CRF [19], but Kober reported that cancer patients with higher education were more likely to experience severe fatigue [20]. This may be ascribed to the possibility that highly educated patients have a higher knowledge level, most of them are engaged in mental labor, face relatively higher pressure, heavier mental burden, higher expectation of disease treatment and knowledge of the disease, all of which increase the level of fatigue. Inconsistencies in findings from previous studies highlight the need for a more nuanced examination of factors influencing educational attainment in future research.

### LS occurrence and risk factors in geriatric cancer patients

LS is one of the major factors contributing to disability in geriatric cancer patients, and their MF continues to decline with age [12]. This study found that the occurrence of LS in geriatric cancer patients was 30.9%, which was lower compared with that of LS in Japanese cancer patients, probably because only cancer patients with high self-care ability and no primary disease of the orthopedic system were included in this study, and the general condition of the subjects was better relative to those in Hirahata’s study [11].

In this study, we found that advanced age, fear of falling, having three or more chronic diseases, and the presence of CRF were the main factors influencing LS in geriatric cancer patients (*P* < 0.05). Table 1 shows that the older the age, the greater the occurrence of LS in geriatric cancer patients (*P* < 0.05). Several age-related changes can increase the risk of developing LS in older adults. These include the gradual decline of the neurophysiological system and postural control, leading to blurred vision, vestibular dysfunction, altered central processing of sensory information, decreased muscle strength, and slowed reaction time [21].

In this study, fear of falling was 58.8%, which exceeded that in the Makino’s study [22]. This may be ascribed to the lack of awareness of falls due to the low literacy level and poor health awareness of elderly oncology patients in China. Elderly oncology patients who are afraid of falling will limit their daily life activities, which is very likely lead to LS, and will cause many forms of psychological trauma, affecting the motor function of geriatric cancer patients [23].

Moreover, the occurrence of LS in geriatric cancer patients was higher in those with 3 or more chronic diseases than in those without chronic diseases (*P* < 0.05), which is consistent with the findings of Iwaya [24]. Geriatric cancer patients with multiple chronic conditions face limitations in their exercise choices. For example, geriatric cancer patients who have suffered a stroke may be restricted in their ability to walk, run, or climb stairs due to impaired balance [25]. Similarly, geriatric cancer patients with cardiovascular or pulmonary diseases may engage in less physical activity or avoid exercise altogether due to compromised heart and lung function [26].

Therefore, to improve the exercise function of our elderly oncology patients, geriatric cancer patients of advanced age should engage in resistance exercise to prevent and control the occurrence of chronic diseases. For individuals with fear of falling, healthcare professionals can provide education on safe and appropriate activities. This can help improve their confidence in movement, leading to better motor function and potentially reducing the risk of developing LS.

### Positive correlation between CRF and LS in geriatric cancer patients

LS is an important indicator of MF, and few studies have explored the association between CRF and LS. Based on the statistical results of this study, a total of 144 individuals were diagnosed with LS, among which 96 (66.7%, 96/144) were both CRF + and LS + , and 48 (33.3%, 48/144) were CRF- and LS + . In contrast, 322 individuals did not have LS, with 108 (33.5%, 108/322) being CRF + and LS-, and 214 (66.5%, 214/322) being CRF- and LS-. These findings indicate that individuals with LS have a higher incidence of CRF, while those without LS have a lower incidence of CRF. Table 3 shows that the occurrence of LS in geriatric cancer patients with CRF was 3.278-fold higher than that of those without cancer-caused fatigue (*P* < 0.05). Table 2 shows that the risk of developing CRF in geriatric cancer patients with LS was 3.351-fold higher compared with that of geriatric cancer patients without LS (*P* < 0.05). Our analysis revealed significant correlations (*P* < 0.05) between total score of GLFS-25 and its sub-scores (pain, daily living activities, social function, and mental health) with CRF scores in geriatric cancer patients. These findings suggest a potential reciprocal relationship between CRF and LS, where each condition may exacerbate the other, creating a cyclical pattern.

This is because LS directly aggravates the decline in MF in cancer patients [12], and according to Ryan’s study, lack of physical activity and decline in MF enhances the occurrence and severity of CRF in cancer patients [27]. Exercise and physical activity are known to improve CRF, the presence of LS might impair activity and therefore cause CRF to be worse [4,10]. In geriatric cancer patients, the effect of LS on MF seems to be more severe due to their old age and coexistence of multiple diseases [11]. Furthermore, CRF can exacerbate LS in geriatric cancer patients. CRF can diminish their response to stimuli, reduce their motivation to engage in activities, and limit their ability to perform daily tasks, all of which can contribute to the development of LS.

The observation that CRF is highly correlated with LS provides an avenue for enhancing QoL intervention in geriatric cancer patients. In clinical practice, LS presents a valuable entry point for research. By focusing on improving LS, healthcare professionals can identify opportunities for intervention, particularly when LS is reversible. This multi-pronged approach could involve addressing pain, enhancing daily living activities, promoting social interaction, and supporting mental well-being. Ultimately, improving LS in geriatric cancer patients can not only directly enhance their mobility and function but also indirectly lead to improvements in their CRF through increased physical activity.

## Conclusion

The occurrence of CRF and LS in geriatric cancer patients is high, and the two are mutually influential and highly correlated (*P* < 0.001). Geriatric cancer patients who comorbid with LS demonstrate a heightened susceptibility to CRF. Geriatric cancer patients who are older, have a fear of falling, suffering from more chronic diseases, and comorbid with CRF demonstrate heightened susceptibility to LS. Healthcare professionals should develop interventions to increase awareness about CRF and LS among geriatric cancer patients. By addressing LS from various aspects, including controlling the number of complications due to advanced old age and other factors, as well as spreading proper fall-related knowledge and controlling the awareness of fear of falling, healthcare professionals can indirectly alleviate CRF symptoms and improve the overall quality of life for these patients.

## Limitations

This study has the following limitations: ① Data were collected from a single-center, and involved a cross-sectional survey. Therefore, future studies should validate our findings in larger populations; ② Patients who were able to undergo active antitumor therapy at our institution were in relatively good basic condition, so the elderly oncology patients in this study were better able to take care of themselves; however, there were still some patients who were more ill or unable to take care of themselves (Barthel Index > 60), and these patients were not included in this study. The CRF and LS status of elderly tumor patients may be more serious in real life. In future studies we will include more geriatric cancer patients with various physical conditions and pay more attention to CRF and LS in patients with poorer MF; ③ This study examined the association between CRF and LS in geriatric cancer patients, without restricting tumor type, staging, or considering specific risk factors such as treatment use and medical history. Future studies will stratify patients by these risk factors to better understand CRF and LS in these subgroups.

## References

[1] Maomao C, He L, Dianqin S, et al. Current cancer burden in China: epidemiology, etiology, and prevention. *Cancer Biol Med*. 2022;19(8):1121-1138. doi: 10.20892/j.issn.2095-3941.2022.0231 PMC9425189 36069534

[2] Qiu H, Cao S, Xu R. Cancer incidence, mortality, and burden in China: a time-trend analysis and comparison with the United States and United Kingdom based on the global epidemiological data released in 2020. Cancer Commun (Lond). 2021;41(10):1037–48. doi: 10.1002/cac2.12197 34288593 PMC8504144

[3] Owusu C, Berger NA. Geriatric management of older cancer patients: A call for action beyond assessments. J Geriatr Oncol. 2019;10(6):845–6. doi: 10.1016/j.jgo.2019.08.007 31474472

[4] Thong MSY, van Noorden CJF, Steindorf K, Arndt V. Cancer-Related Fatigue: Causes and Current Treatment Options. Curr Treat Options Oncol. 2020;21(2):17. doi: 10.1007/s11864-020-0707-5 32025928 PMC8660748

[5] Ma Y, He B, Jiang M, Yang Y, Wang C, Huang C, et al. Prevalence and risk factors of cancer-related fatigue: A systematic review and meta-analysis. Int J Nurs Stud. 2020;111 103707. doi: 10.1016/j.ijnurstu.2020.103707 32920423

[6] Al Maqbali M. Cancer-related fatigue: an overview. Br J Nurs. 2021;30(4):S36–43. doi: 10.12968/bjon.2021.30.4.S36 33641391

[7] Arring NM, Barton DL, Brooks T, Zick SM. Integrative Therapies for Cancer-Related Fatigue. Cancer J. 2019;25(5):349–56. doi: 10.1097/PPO.0000000000000396 31567463 PMC7388739

[8] Soones T, Ombres R, Escalante C. An update on cancer-related fatigue in older adults: A narrative review. J Geriatr Oncol. 2022;13(2):125–31. doi: 10.1016/j.jgo.2021.07.006 34353750

[9] Kobayashi T, Morimoto T, Otani K, Mawatari M. Locomotive Syndrome and Lumbar Spine Disease: A Systematic Review. J Clin Med. 2022;11(5):1304. doi: 10.3390/jcm11051304 35268395 PMC8911455

[10] Akahane M, Maeyashiki A, Tanaka Y, Imamura T. The impact of musculoskeletal diseases on the presence of locomotive syndrome. Mod Rheumatol. 2019;29(1):151–6. doi: 10.1080/14397595.2018.1452173 29529893

[11] Hirahata M, Imanishi J, Fujinuma W, Abe S, Inui T, Ogata N, et al. Cancer may accelerate locomotive syndrome and deteriorate quality of life: a single-centre cross-sectional study of locomotive syndrome in cancer patients. Int J Clin Oncol. 2023;28(4):603–9. doi: 10.1007/s10147-023-02312-2 36806698 PMC9939082

[12] Kawano H, Hirahata M, Imanishi J. Locomotive syndrome in cancer patients: a new role of orthopaedic surgeons as a part of comprehensive cancer care. Int J Clin Oncol. 2022;27(8):1233–7. doi: 10.1007/s10147-022-02194-w 35690700 PMC9309135

[13] Fabi A, Bhargava R, Fatigoni S, Guglielmo M, Horneber M, Roila F, et al. Cancer-related fatigue: ESMO Clinical Practice Guidelines for diagnosis and treatment. Ann Oncol. 2020;31(6):713–23. doi: 10.1016/j.annonc.2020.02.016 32173483

[14] Mahnoey F I, Barthel D W. Functional Evaluation: The Barthel Index[J].Md State Med J,1965,14:61-5. 14258950

[15] Okuyama T, Akechi T, Kugaya A, Okamura H, Shima Y, Maruguchi M, et al. Development and validation of the cancer fatigue scale: a brief, three-dimensional, self-rating scale for assessment of fatigue in cancer patients. J Pain Symptom Manage. 2000;19(1):5–14. doi: 10.1016/s0885-3924(99)00138-4 10687321

[16] Seichi A, Hoshino Y, Doi T, Akai M, Tobimatsu Y, Iwaya T. Development of a screening tool for risk of locomotive syndrome in the elderly: the 25-question Geriatric Locomotive Function Scale. J Orthop Sci. 2012;17(2):163–72. doi: 10.1007/s00776-011-0193-5 22222445

[17] Pearson EJM, Morris ME, McKinstry CE. Cancer related fatigue: implementing guidelines for optimal management. BMC Health Serv Res. 2017;17(1):496. doi: 10.1186/s12913-017-2415-9 28720109 PMC5516360

[18] Vadiraja HS, Rao RM, Nagarathna R, Nagendra HR, Patil S, Diwakar RB, et al. Effects of Yoga in Managing Fatigue in Breast Cancer Patients: A Randomized Controlled Trial. Indian J Palliat Care. 2017;23(3):247–52. doi: 10.4103/IJPC.IJPC_95_17 28827926 PMC5545948

[19] Bower JE, Ganz PA, Aziz N, Fahey JL. Fatigue and proinflammatory cytokine activity in breast cancer survivors. Psychosom Med. 2002;64(4):604–11. doi: 10.1097/00006842-200207000-00010 12140350

[20] Kober KM, Smoot B, Paul SM, Cooper BA, Levine JD, Miaskowski C. Polymorphisms in Cytokine Genes Are Associated With Higher Levels of Fatigue and Lower Levels of Energy in Women After Breast Cancer Surgery. J Pain Symptom Manage. 2016;52(5):695-708.e4. doi: 10.1016/j.jpainsymman.2016.04.014 27664835 PMC5107347

[21] Sorond FA, Cruz-Almeida Y, Clark DJ, Viswanathan A, Scherzer CR, De Jager P, et al. Aging, the Central Nervous System, and Mobility in Older Adults: Neural Mechanisms of Mobility Impairment. J Gerontol A Biol Sci Med Sci. 2015;70(12):1526–32. doi: 10.1093/gerona/glv130 26386013 PMC4643615

[22] Makino K, Makizako H, Doi T, Tsutsumimoto K, Hotta R, Nakakubo S, et al. Fear of falling and gait parameters in older adults with and without fall history. Geriatr Gerontol Int. 2017;17(12):2455–9. doi: 10.1111/ggi.13102 28656737

[23] Aburub AS, P Phillips S, Curcio C-L, Guerra RO, Auais M. Fear of falling in community-dwelling older adults diagnosed with cancer: A report from the International Mobility in Aging Study (IMIAS). J Geriatr Oncol. 2020;11(4):603–9. doi: 10.1016/j.jgo.2019.09.001 31653454

[24] Iwaya T, Doi T, Seichi A, Hoshino Y, Ogata T, Akai M. Characteristics of disability in activity of daily living in elderly people associated with locomotive disorders. BMC Geriatr. 2017;17(1):165. doi: 10.1186/s12877-017-0543-z 28747158 PMC5527391

[25] Saunders DH, Sanderson M, Hayes S, Johnson L, Kramer S, Carter DD, et al. Physical fitness training for stroke patients. Cochrane Database Syst Rev. 2020;3(3):CD003316. doi: 10.1002/14651858.CD003316.pub7 32196635 PMC7083515

[26] Inoue S, Shibata Y, Kishi H, Nitobe J, Iwayama T, Yashiro Y, et al. Decreased left ventricular stroke volume is associated with low-grade exercise tolerance in patients with chronic obstructive pulmonary disease. BMJ Open Respir Res. 2017;4(1):e000158. doi: 10.1136/bmjresp-2016-000158 28176968 PMC5278312

[27] Marker RJ, Wechsler S, Leach HJ. Cancer-related fatigue is associated with objective measures of physical function before and after a clinical exercise program: A retrospective analysis. Rehabil Oncol. 2024;42(1):31–8. doi: 10.1097/01.reo.0000000000000354 38774708 PMC11104554

